# Using Gesture to Facilitate L2 Phoneme Acquisition: The Importance of Gesture and Phoneme Complexity

**DOI:** 10.3389/fpsyg.2020.575032

**Published:** 2020-11-23

**Authors:** Marieke Hoetjes, Lieke van Maastricht

**Affiliations:** Centre for Language Studies, Radboud University, Nijmegen, Netherlands

**Keywords:** second language acquisition, phonemes, audiovisual, deictic gesture, iconic gesture, accentedness, comprehensibility

## Abstract

Most language learners have difficulties acquiring the phonemes of a second language (L2). Unfortunately, they are often judged on their L2 pronunciation, and segmental inaccuracies contribute to miscommunication. Therefore, we aim to determine how to facilitate phoneme acquisition. Given the close relationship between speech and co-speech gesture, previous work unsurprisingly reports that gestures can benefit language acquisition, e.g., in (L2) word learning. However, gesture studies on L2 phoneme acquisition present contradictory results, implying that both specific properties of gestures and phonemes used in training, and their combination, may be relevant. We investigated the effect of phoneme and gesture complexity on L2 phoneme acquisition. In a production study, Dutch natives received instruction on the pronunciation of two Spanish phonemes, /u/ and /θ/. Both are typically difficult to produce for Dutch natives because their orthographic representation differs between both languages. Moreover, /θ/ is considered more complex than /u/, since the Dutch phoneme inventory contains /u/ but not /θ/. The instruction participants received contained Spanish examples presented either via audio-only, audio-visually without gesture, audio-visually with a simple, pointing gesture, or audio-visually with a more complex, iconic gesture representing the relevant speech articulator(s). Preceding and following training, participants read aloud Spanish sentences containing the target phonemes. In a perception study, Spanish natives rated the target words from the production study on accentedness and comprehensibility. Our results show that combining gesture and speech in L2 phoneme training can lead to significant improvement in L2 phoneme production, but both gesture and phoneme complexity affect successful learning: Significant learning only occurred for the less complex phoneme /u/ after seeing the more complex iconic gesture, whereas for the more complex phoneme /θ/, seeing the more complex gesture actually hindered acquisition. The perception results confirm the production findings and show that items containing /θ/ produced after receiving training with a less complex pointing gesture are considered less foreign-accented and more easily comprehensible as compared to the same items after audio-only training. This shows that gesture can facilitate task performance in L2 phonology acquisition, yet complexity affects whether certain gestures work better for certain phonemes than others.

Preliminary versions of parts of this paper were presented at the International Congress of Phonetic Sciences in August 2019 in Melbourne, Australia (Van Maastricht et al., 2019), at the 29th conference of the European Second Language Association in August 2019 in Lund, Sweden (Hoetjes et al., 2019b), and at the Gesture and Speech in Interaction conference in September 2019 in Paderborn, Germany (Hoetjes et al., 2019a). The current paper includes a more detailed theoretical background, description of the experimental methods, and discussion of the findings, as well as more advanced statistical analyses over the complete data set in the case of Study I and analyses over a new data set in the case of Study II.

## Introduction

Human communication is multimodal: When people communicate face-to-face, they do not only use speech but also various non-verbal communicative cues, such as facial expressions and hand gestures. In this study, we focus on one of these aspects of non-verbal communication, namely co-speech hand gestures, within the context of foreign language learning. There is general agreement in the literature that speech and co-speech gestures are closely related and that they are integrated in various ways (McNeill, 1992; Kendon, 2004; Wagner et al., 2014). This is apparent, for example, by the fact that there is a close temporal and semantic coordination between speech and gesture. This means that roughly speaking, speech and gesture tend to express the same thing at the same time (see, Gullberg, 2006, for an overview). Moreover, the integration between speech and gesture is reflected in the parallel development of the two modalities: For instance, in first language (L1) acquisition, it has been shown that gestures play a facilitating role in vocabulary learning in children, with gesture production predicting their subsequent lexical and syntactic development (e.g., Goldin-Meadow, 2005). Both modalities have also been shown to break down in a parallel way, for example during disfluencies (e.g., Seyfeddinipur, 2006; Graziano and Gullberg, 2018) or as a result of aphasia (Van Nispen et al., 2016). In short, the relationship between speech and gesture plays a crucial role in our communicative processes. Given this close relationship between speech and gesture in communication, the possible benefit of gesture in learning contexts has been a topic of research in different scientific fields, one of which is second language (L2) acquisition. While gesture is often intuitively used by teachers in classrooms (cf. Smotrova, 2017), very little is known about the specifics of the interplay between both modalities in a learning context. Hence, in the current study, we compare the use of different types of gestures in the context of L2 phoneme acquisition to determine in which way gesture and phoneme complexity in L2 training affect the phoneme productions of Dutch learners of Spanish (Study I) and the perceptions of Spanish natives with respect to these non-native productions (Study II). Before turning to the specifics of our research, we first review the relevant literature.

### Multimodality in Learning Contexts

Gesture can play a facilitative role in various kinds of learning situations. For example, previous work has shown that students take teachers’ gestures into account and that teachers can thus use gesture to help students learn mathematical concepts (e.g., Goldin-Meadow et al., 1999; Yeo et al., 2018). Focusing on L2 learning, various studies have shown that gestures can play a facilitative role in the acquisition of L2 vocabulary, both by children and adults. Tellier (2008), for example, had 5-year old French children learn English words associated with either a picture or a gesture and found that the gesture group did better than the picture group. For adults, Kelly et al. (2009) likewise found that when novel Japanese words were presented to native speakers of English, they were better at learning these words when they were presented with hand gestures, as compared to without hand gestures. In these studies, iconic gestures were used, which have a clear semantic relationship to the lexical items they accompany. The conclusion we can draw from these findings is that presenting semantic information in several modalities strengthens learners’ memory of the words’ semantic meaning (e.g., Tellier, 2008; Kelly et al., 2009; Macedonia et al., 2011).

Apart from vocabulary acquisition, it is important for L2 learners to also learn how to correctly pronounce the sounds of their target language. On the one hand, phoneme acquisition is one of the aspects of L2 acquisition learners generally find most difficult (see, e.g., collected papers in Bohn and Munro, 2007), while on the other hand, an atypical pronunciation is an aspect of speech that is very salient to native listeners (see Derwing and Munro, 2009 and the references therein), even if it doesn’t necessarily affect their perceived ease of comprehensibility or actual processing of the L2 speech (Munro and Derwing, 1999; Van Maastricht et al., 2016). Moreover, pronunciation is often one of the aspects of the L2 that learners are eager to acquire since most of them aim to sound as native-like as possible in the L2 (Timmis, 2002; Derwing, 2003). A native-like pronunciation is especially important given that a clear non-native pronunciation has been shown to negatively affect the way speakers are perceived (Lev-Ari and Keysar, 2010) and segmental inaccuracies contribute to miscommunication (Caspers and Horłoza, 2012).

Given the tight relationship between speech and gesture and the fact that gestures can facilitate L1, and even L2, development, it is not such a strange idea that gesture may also play a role in L2 phoneme acquisition. Anecdotally, L2 teachers report to regularly use gestures in the classroom when teaching different aspects of L2 phonology but there are also empirical reasons to assume that gestures could play a facilitative role in L2 phoneme acquisition even though, to date, most research on multimodal L2 phonology acquisition has not focused on gestures. For instance, Hazan et al. (2005) have shown that multimodal training on English phoneme contrasts, in this case through the auditory modality only as compared to through the audiovisual modality, generally benefitted the production and perception of L2 phonemes by Japanese learners of English. Hardison (2003) reports similar results with Japanese and Korean intermediate-level learners of English and found that improvement in phoneme perception also led to improved phoneme production, which she attributes to the fact that the audiovisual training leaves multiple memory traces, while the auditory training only left one.

Using a form of multimodal training that is similar to a gesture, Zhang et al. (2020) studied the facilitative effect of hand-clapping on L2 pronunciation. They showed that French words produced by Chinese adolescents were rated as marginally more nativelike after they had seen and reproduced training videos in which the speaker clapped to visualize the rhythmic structure of the French words as compared to seeing a speaker that did not move her hands and not moving their own hands. They also found a significant effect of training condition on final syllable duration, reflecting the final stress placement that is typical of French, with longer final syllable lengths for items produced after the clapping condition. Like hand-clapping, gestures are not only visual but also consist of movements. Hence, these previous findings would suggest that using gesture in language training, as opposed to using only auditory input or visual input without movements, could facilitate L2 phoneme acquisition. Indeed, some previous studies have been conducted specifically on the role of gestures in the acquisition of L2 tonal and phonemic contrasts. However, the results of these studies are inconclusive.

### Gesture and L2 Phonology

On the one hand, there is previous work suggesting that gestures can indeed play a role in the acquisition of certain aspects of L2 phonology, such as the perception of L2 tones and intonation contours. Kelly et al. (2017) conducted a study in which native speakers of English listened to different types of Japanese phonemic contrasts. The speech sounds contrasted concerning their vowel length or their sentence-final intonation. Participants were presented with training on the relevant phonemic differences, followed by videos showing either speech without gestures, speech with congruent metaphoric gestures visualizing the contrast, where the gestures’ meaning was in line with the phonemic meaning (short vs. long vowel, or rising vs. falling intonation), or speech with incongruent gestures (e.g., a short vowel with a long gesture). After each video, participants had to indicate whether they perceived the audio to contain a long vs. short vowel, or rising vs. falling intonation. Although results were not clear-cut for the vowel length contrasts, congruent gestures did help to correctly perceive intonational contrasts, as compared to incongruent gesture or no gesture conditions. In a similar vein, work by Hannah et al. (2017) on Mandarin tones used speech-accompanying congruent and incongruent metaphoric gestures and found that perceivers often relied on the visual cues they received, which in the case of incongruence between speech and gesture resulted in participants incorrectly perceiving what they had heard. Gluhareva and Prieto (2017) did not use metaphoric gestures but beat gestures, and showed that viewing beat gestures during discourse prompts improved L2 pronunciation, as measured by accentedness ratings by English natives of short stories produced by Catalan learners of English. Moreover, recent work by Li et al. (2020) focused on the L2 acquisition of Japanese vowel-length contrasts and although they found that gesture (versus no gesture) did not improve L2 vowel length perception, gesture did facilitate correct L2 vowel length production.

On the other hand, there has been work suggesting that gestures do not play a facilitative role in the acquisition of some aspects of L2 phonology, such as the perception of phonemic vowel length distinctions in Kelly et al. (2017), where viewing gestures did not facilitate the perception of phonemic vowel length distinctions. Several other studies also did not report positive effects of gesture on L2 phoneme perception. For instance, in work by Kelly et al. (2014) and by Hirata et al. (2014), the L2 acquisition of phonemic vowel length contrasts was investigated by letting English naïve learners of Japanese observe or also produce gestures related to the syllable or the mora structure of the target word. In an auditory identification task, no differences between the training conditions were found. The authors suggest that this could mean that gestures are not suited for learning phonetic distinctions^1^. Earlier work by Kelly and Lee (2012) expounds this point of view somewhat by stating that gesture may help in acquiring phonetically easy phonemic contrasts, but hinders the acquisition of phonetically hard contrasts because iconic gestures could add too much semantic content to the spoken input, which complicates the acquisition of new phonemes since the learner is simultaneously paying attention to the novel sounds and the contents of the gesture. Hence, they suggest that “it is possible that gesture facilitates local processing of speech sounds only for familiar phonemes in one’s native language” (p. 804), which is a relevant factor in the present study.

This contrast between gestures playing a facilitative role in certain contexts but hindering L2 acquisition in others has, in some cases, even been shown within studies. As discussed above, Kelly et al. (2017), for example, showed that similar metaphoric gestures helped for perceiving non-native intonation contours, but did not help in perceiving vowel length differences. Likewise, Morett and Chang (2015) studied the acquisition of L2 Mandarin lexical tone perception by English learners and found that gestures that visualize the target pitch contour helped acquisition, while gestures referring to the semantic meaning of the word hindered correct tone identification. Clearly, the role of gestures in the L2 acquisition of phonemes is not straight-forward. As prior studies used varying research methods and focused on different aspects of L2 phonology, it remains unclear whether the contradictory findings within the field of L2 phonology acquisition are due to methodological discrepancies or to the fact that the specific properties of the gestures used in training, as well as the properties of the phonetic feature to be acquired, contribute to the effectiveness of the use of gesture in L2 pronunciation training. It has been suggested (Kelly et al., 2014) that using gestures for complex L2 input, for example, because the learner has a low proficiency or because the contrast in question is hard to acquire, may hinder rather than help acquisition. In those cases, the processing resources needed for the interpretation of the speech might be prioritized to those needed to process the gesture. This would be in contrast with easy L2 acquisition contexts, where gestures that may play a beneficial role can be processed alongside speech. In any case, the lack of agreement between the different studies in this domain means that it is hard to draw clear conclusions, and indeed, Kelly et al. (2017, p. 1) suggest that “gestures help with some –but not all- novel speech sounds in a foreign language.”

### The Present Study

What most previous studies on L2 phoneme acquisition have in common is that they generally focus on learners’ *perception* skills, that is, whether certain types of language training result in learners being able to recognize or distinguish between different phonemes. In most cases, we do not yet know to what extent these results can be extended to learners’ *production* of L2 phonemes. In other words, can a certain type of training result in L2 learners’ improved ability to pronounce the phonemes in the L2? Hence, one of the goals of this study is to focus on L2 phoneme production. Also, one potential reason for the diverging findings in previous work is that the effect of gestures in L2 phoneme training on L2 phoneme perception has been investigated using various types of gestures and hand movements, but without directly comparing them. Studies have, for example, looked at the use of beats (Gluhareva and Prieto, 2017), which are simple rhythmical gestures, but also at, arguably more complex, metaphoric gestures (Kelly et al., 2014, 2017), which are like iconic gestures in the sense that they show a clear semantic relationship between the movement and the content of speech, but are produced during abstract speech. We are unaware of previous work incorporating deictic (i.e., pointing) gestures in L2 acquisition or of work on L2 phoneme acquisition comparing the effect of different types of gestures. These differences between studies make it hard to draw clear conclusions about the educational value of different types of gestures. Differences in the speech-gesture relationship between types of gestures mean that their potential role in L2 acquisition is not self-evident. Hence, another goal of this study is to compare different *types of gestures* and the role they may play in the acquisition of L2 phoneme production.

In the current study, we thus aim to investigate whether different types of gestures can facilitate L2 learners’ productions of two different L2 phonemes, which vary in complexity. We do so by conducting two experimental studies. In our production task (Study I), we provide Dutch learners of Spanish with training on two phonemes that are typically difficult for them: /u/ and /θ/. We have chosen to approach L2 phoneme acquisition within the context that will likely be typical for adult L2 learners: They usually learn the L2 in a classroom setting and, in contrast to infants, are generally able to read, which means they often receive a large part of their instruction from written textbooks and exercises and part of the challenge lies therefore in making the correct association between spelling and sound. This means that producing the right L2 phoneme is not only dependent on whether they are familiar with the sound itself but also on whether they are accustomed to relating that particular sound to the correct grapheme. Prior research (e.g., Escudero et al., 2014) has shown that stimuli with incongruent grapheme-orthography mapping hinder L2 performance in various areas. We employed this distinction in order to manipulate phoneme complexity in our study: While there are subtle phonetic differences between the production of /u/ in Dutch and Spanish, it is a segment that is present in both the Dutch and the Spanish phoneme inventory. The difficulty for Dutch learners of Spanish lies in the fact that, in Spanish, the phoneme that corresponds to the grapheme < u > is always /u/, whereas in Dutch several phonemes correspond to the grapheme < u >, for instance, /æ/ as in *dun* (“thin”), /y/ as in *pure* (“pure”) and in combination with other vowels there is even more variation possible with realizations, for instance, as /æy/, /ø/, or /ɑu/, as in *muis* (“mouse”), *leuk* (“fun”), or *rauw* (“raw,” Kooij and Van Oostendorp, 2003). Conversely, when it comes to the acquisition of /θ/, the challenge is 2-fold: not only is /θ/ not a part of the Dutch phoneme inventory^2^ and thus a new segment for which a category needs to be created, its only corresponding grapheme in Spanish is >Z<,^3^ while in Dutch >Z< is typically pronounced as /z/ or /s/. In sum, while /u/ requires a novel grapheme to phoneme correspondence, /θ/ requires both a novel grapheme to phoneme correspondence and the creation of a new category in the phoneme inventory. These differences between /u/ and /θ/ allow us to manipulate phoneme complexity in our production task.

Our Dutch learners of Spanish received instruction on /u/ and /θ/ in one of four conditions: audio-only (AO), audio-visual (AV), audio-visual with a pointing gesture (AV-P), or audio-visual with an iconic gesture (AV-I). The AO condition serves as a baseline, to which we will compare the other conditions, of which the latter two contain either a less or more complex gesture: A pointing gesture was chosen as a less complex gesture, as it has no intrinsic semantic meaning and only serves to draw the listeners attention to a specific feature in the context, in our case, the mouth of the native speaker of Spanish pronouncing an example item. An iconic gesture was chosen as a more complex gesture, as it does have intrinsic semantic meaning because it illustrates to the listener which articulator is involved in the production of the target sounds and in which way it should be used. Our analyses will focus on whether gesture complexity and phoneme complexity affect the production of the target phonemes by Dutch learners of Spanish. In a perception task (Study II), Spanish natives listened to words containing the target phonemes that were produced by the Dutch learners of Spanish before and after AV, AV-P, or AV-I training and judged them on foreign accentedness and comprehensibility.

Based on previous studies (Hardison, 2003; Hazan et al., 2005), we hypothesize that adding audio-visual information to L2 phoneme training will facilitate phoneme acquisition, as compared to providing only audio information. Given that some previous work (e.g., Hannah et al., 2017; Kelly et al., 2017) has shown that gestures can be helpful in the acquisition of certain phonemes, we expect that including gestures in language training will be more beneficial than not including them, but possibly only in a context that is less cognitively demanding, that is, when producing /u/, but not /θ/ (Kelly and Lee, 2012). This would be in line with an embodied approach to cognition, which implies that not only performing but also seeing gestures benefits memory performance, which is essential in our phoneme production task (Madan and Singhal, 2012). Finally, given the lack of previous work that directly compares the role of different types of gestures in language acquisition, we cannot predict different effects between different types of gestures, but we speculate that there might be a difference between the potential facilitative effect of deictic and iconic gestures, based on the cognitive resources needed to process them. If this indeed affects their effectiveness in L2 pronunciation training, one would expect that pointing gestures might be more helpful than iconic gestures, which would be more cognitively demanding and thus entail less processing resources available for the perception and acquisition of the phoneme itself.

## Study I

### Method

#### Participants

In study I, 50 native speakers of Dutch, who did not speak any Spanish, took part. They were 28 women and 22 men, with a mean age of 25 years old (range 18–61 years old). Participants had no auditory or visual impairments that could affect their participation. Participants were recruited via the Radboud University research participation system and received either credits or a small financial reward for taking part.

#### Design

Study I consisted of a pretest – training – posttest paradigm. We used a between-subjects design in which participants took part in one of four experimental training conditions: AO (*n* = 12), AV (*n* = 13), AV-P (*n* = 13), or AV-I (*n* = 12). The dependent variable was the pronunciation of the target phonemes, coded as either on-target or not.

#### Materials

##### Sentences

**FIGURE 1:**
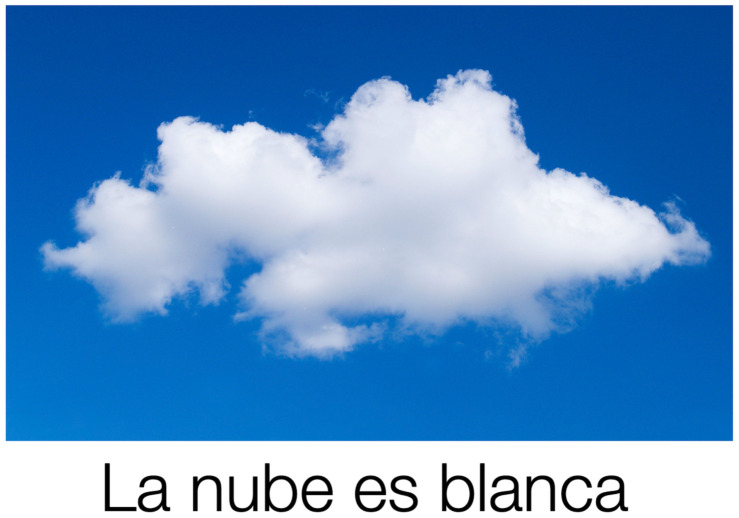
Example of an experimental item containing the target phoneme /u/.

In the pretest and the posttest, participants read out loud 16 Spanish four-word sentences (in one of two randomized orders) that were easy to parse, half of which were experimental items. In each experimental item, the first syllable of the two-syllable noun in the sentence contained either /u/ or /θ/ (e.g., *La nube es blanca, la zeta es verde)*. Each of the two target phonemes occurred in four target words, for /u/: *muro, nube, ruta, suma*; for /θ/: *zeta, zorro, zueco, zumo*. The eight remaining filler items also contained the target phonemes, but at different positions within the words or the sentence. The filler items were not analyzed. The target phonemes were embedded in the four-word sentences and presented to participants one at a time on PowerPoint slides. Each written sentence was accompanied by a picture illustrating the meaning of the sentence (see Figure 1). This was done to make the task more interesting and to help participants understand the semantic meaning of the sentence.

##### Training

**FIGURE 2:**
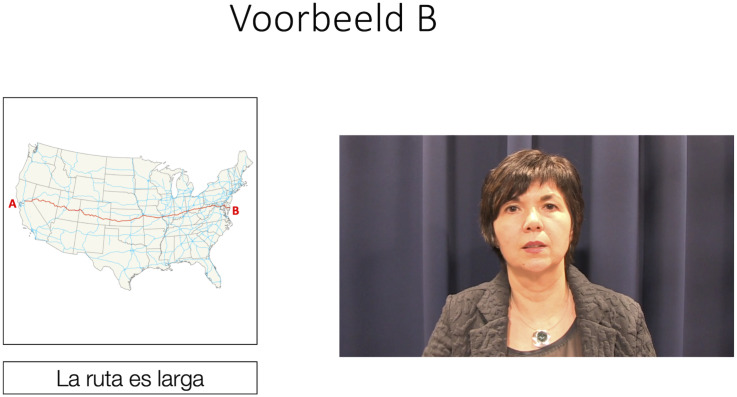
Example of slide illustrating phoneme pronunciation within a sentence; screenshot of video on the right, sentence pronounced by the native speaker on the left, with accompanying picture.

After the pretest and before the posttest, participants received training on how to pronounce the target phonemes /θ/ and /u/ (in counterbalanced order) in Spanish. This training consisted of a set of three PowerPoint slides for each phoneme. On the first slide, written information was given on how to pronounce the target phoneme. Specifically, participants were told that the Spanish pronunciation of both graphemes differs from the Dutch pronunciation of these graphemes. Moreover, participants were explicitly instructed which articulatory gestures are necessary for nativelike pronunciation (i.e., “when pronouncing the letter “u” in Spanish, you need to round your lips” and “when pronouncing the letter “z” in Spanish, you need to place your tongue between your teeth and push out the air”). Apart from the written text, participants were also given an example of a native speaker of Spanish pronouncing the target phoneme in isolation. On the two following slides, participants were given two examples of the pronunciation of the target phoneme embedded within an example sentence. These examples (all produced by the same native speaker of Spanish) were accompanied by the written sentence and a picture illustrating the meaning of the sentence, in the same way as during the pretest and posttest (see Figure 2). The training was self-paced and participants took roughly 3 to 4 min to complete it. They were free to listen to/view the example fragments as many times as they wanted.

**FIGURE 3:**
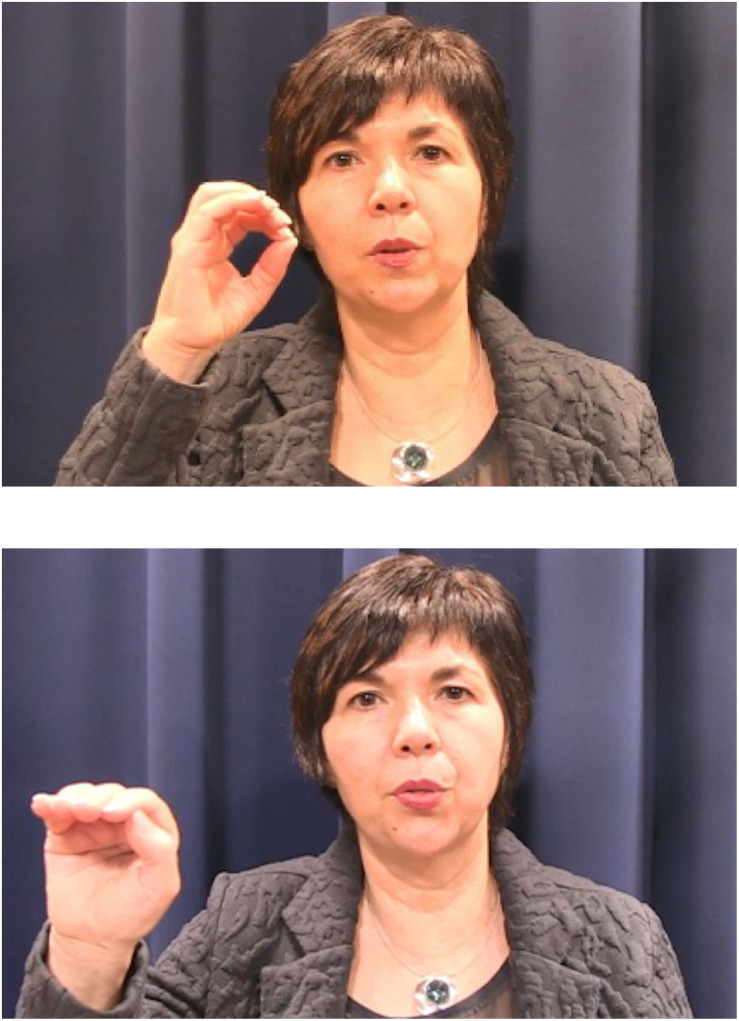
Stills from training video in AV-I condition showing the articulatory gesture needed for /u/ (top still) and /θ/ (bottom still).

To manipulate training condition, the visual information given in the examples during the training varied, while the same audio was dubbed over all conditions. In the AO condition, participants heard the audio examples but did not see any video recordings of the speaker. In the AV condition, participants saw a video clip of the speaker producing the examples, but the speaker did not move her hands. In the AV-P condition, participants saw videos in which the speaker produced a pointing gesture toward her mouth while she produced the target phoneme. In the AV-I condition, participants saw the speaker produce an iconic gesture while she produced the target phoneme (see Figure 3 for examples). This iconic gesture represented the articulatory gesture needed for on-target phoneme production, as was explained verbally on the first training slide. For /u/, the iconic gesture was a one-handed gesture representing the rounding of the lips, and for /θ/, the iconic gesture was a one-handed gesture indicating that the speaker should push their tongue between their teeth. Both iconic gestures were made with one hand, roughly equally complex with respect to finger configuration, and not necessarily representing all articulators in the gesture but only the most relevant one for the learner. In the case of /θ/, Dutch learners of Spanish are familiar with non-sibilant fricatives (e.g., /f/ and /v/) but not interdental ones, so they need to know that they should push their tongue out of their mouth, which is only possible by placing it in between the teeth and lips. Concerning /u/, Dutch learners of Spanish need to know that correct pronunciation requires a stronger rounding of the lips than needed for any of the Dutch vowels. We performed a posttest for our stimuli among 42 native speakers of Dutch in which we compared the iconic and pointing gestures used for both phonemes with respect to how useful they found the gesture in the context of the L2 training for that specific phoneme, how intuitive they found the gesture in that context and whether they thought they understood why the gesture was chosen in that context. No significant differences were found between gesture type conditions or phoneme conditions for any of our measures, nor did the test reveal any significant interactions. This suggests that any differences between the iconic gestures concerning the way they visualize the relevant articulator did not affect our results.

#### Procedure

To minimize distractions for the participants, the experiment took place in a soundproof booth. The language used throughout the experiment, except for the Spanish sentences during pretest, training, and posttest was Dutch. After participants had received instructions and signed a consent form, they were recorded while they read the 16 Spanish sentences out loud into a microphone (pretest). The pretest was first followed by a language background questionnaire, and then by one of the four types of pronunciation training. After the pronunciation training, participants were again recorded while they read out loud the same 16 Spanish sentences in a reordered version (posttest). Both the pretest and posttest were self-paced and participants were invited to repeat the sentences until they were satisfied with their pronunciation. The last production of each sentence was used for analysis. After completing all tasks, participants were debriefed.

### Results

**FIGURE 4:**
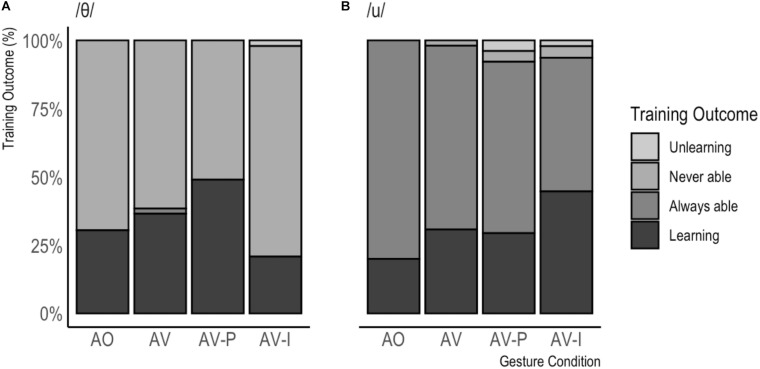
Training Outcome in percentages, separated by Gesture Condition for /θ/ **(A)** and /u/ **(B)**.

The audio recorded during the pretest and the posttest was annotated using Praat (Boersma and Weenink, 2018) concerning the production of the target phonemes. Two phonetically trained coders annotated the 1600 target phonemes (50 speakers × 16 sentences × 2 testing moments), and distinguished between a nativelike production (i.e., as a native speaker of Iberian Spanish would do) and several non-nativelike productions that are typical for native speakers of Dutch (for /θ/, these were /s/, /z/, or “other”; for /u/, these were /y/, /ə/, /Y/, or “other”). In the current analyses, nativelike productions were distinguished from the various non-nativelike productions, collapsing over the various non-target options. There was an overlap of 50% in coding and a good inter-rater reliability (K = 0.900, *p* < 0.001). Productions of target phonemes from the same sentences were compared between the pretest and the posttest, resulting in four different outcome options: (1) the participant was able to produce the target phoneme in the pretest, but not anymore at the posttest; (2) the participant was not able to produce the target phoneme at either the pretest or the posttest; (3) the participant was able to produce the target phoneme both at the pretest and at the posttest; (4) the participant was unable to produce the target phoneme at the pretest, but able to do so at the posttest. Figure 4 and Table 1 summarize the results per learning outcome separated by gesture condition and phoneme. In Table 1, the results are presented in terms of raw frequencies, while percentages are presented in Figure 4. First, we will inspect the data descriptively, followed by inferential statistics in the form of a mixed effects logistic regression analysis in which we distinguished between cases of “learning” (i.e., option 4), and “no learning” (i.e., collapsing options 1–3).

**TABLE 1:** Frequency of Training outcomes for /u/ and /θ/, separated by training condition.

Training Condition	Learning		Always Able		Never Able		Unlearning		Total
	/u/	/θ/	/u/	/θ/	/u/	/θ/	/u/	/θ/	
AO	**9**	**14**	36	0	0	32	0	0	91
AV	**16**	**19**	35	1	1	32	0	0	104
AV-P	**15**	**25**	32	0	2	26	2	0	102
AV-I	**21**	**10**	23	0	2	37	1	1	95
Total	**61**	**68**	126	1	5	127	3	1	392^4^

*The target outcome is printed in bold.*

When inspecting the raw data per training condition in the cases that learning occurred, the Dutch learners of Spanish, in general, appear to benefit from receiving both auditory and visual information. For both phonemes, the cases of learning increase as more visual information is added, except for in the AV-I condition: While the L2 learners who aimed to produce a /u/ benefitted most from seeing an iconic gesture during training, the participants who aimed to produce a /θ/ appeared to benefit most from seeing a pointing gesture.

We used R (version 3.6.1, RCoreTeam, 2019) and the *lme4* package (Bates et al., 2015) to conduct a linear mixed effects logistic regression analysis to model binary outcome variables. The theoretically relevant predictors Gesture Condition (AO, AV, AV-P, or AV-I) and Phoneme (/u/ or /θ/) were included as fixed factors, and Training Outcome (Learning or No Learning) served as the response variable. Random intercepts were added for Speaker and Item. Adding random slopes resulted in models that either failed to converge or had inferior fit. Significance was assessed via likelihood ratio tests comparing the full model to a model lacking only the relevant effect. The complete model provided the best fit as determined by the Akaike Information Criterion, see Table 2 for a complete overview of all effects and coefficients.

**TABLE 2:** Estimated effects and coefficients for Training Outcome.

Learning vs. Not Learning	*β* estimate	Std. error	*z* value	*p* value	95% CI for Odds Ratio		
					Lower	Odds Ratio	Upper
Intercept	−2.06	0.68	−3.06	0.002	0.03	0.13	0.48
Gesture Condition_AV_	0.86	0.77	1.12	0.263	0.52	2.37	10.74
Gesture Condition_AV–P_	0.90	0.76	1.19	0.236	0.55	2.47	11.01
**Gesture Condition_AV–I_**	**1.79**	**0.77**	**2.32**	**0.020**	**1.32**	**6.01**	**27.33**
Phoneme_/θ/_	0.88	0.73	1.20	0.232	0.57	2.41	10.14
Phoneme_/θ/_ * Gesture Condition_AV_	−0.44	0.76	−0.59	0.558	0.15	0.64	2.82
Phoneme_/θ/_ * Gesture Condition_AV–P_	0.23	0.73	0.32	0.753	0.30	1.26	5.30
**Phoneme_/θ/_** * **Gesture Condition_AV–I_**	−**2.30**	**0.78**	−**2.95**	**0.003**	**0.02**	**0.10**	**0.46**

**Random effects**	**Variance**			**Standard deviation**			

Speaker	1.593			1.262			
Item	0.423			0.650			

The intercept represents the following combination of variable levels: Gesture Condition = AO, Phoneme = /u/. Asterisks (*) represent interactions, subscript signals the level of a categorical variable. Significant *p*-values are printed in bold. The model used in this analysis can be described as *Training Outcome ∼ Gesture Condition* × *Phoneme + (1| participant) + (1| item)*.

The analysis revealed that the condition that the participant was assigned to significantly predicted whether learning occurred or not but only when comparing the AV-I condition to the AO condition (the baseline condition, β = 1.79, *p* < 0.05). As gesture condition changes from AO to AV-I, the change in the odds of learning (rather than not learning) is 6.01. In other words, in general, a participant is more likely to learn than not in the AV-I condition than in the AO condition. In addition, there was an interaction between Phoneme and Gesture Condition (β = −2.30, *p* < 0.01), suggesting that the success of being in the AV-I condition depended on whether the participant aimed to produce a /u/ or a /θ/. The odds ratio tells us that as the gesture condition changes from AO to AV-I in combination with the phoneme being produced being a /θ/ instead of a /u/, the change in the odds of learning compared to not learning was 0.10. In order words, as the phoneme that is produced is /θ/ instead of /u/, participants are less likely to learn in the AV-I condition.

### Interim Discussion

In summary, Study I showed that, in general, adding audio-visual information to phoneme pronunciation training aided target-like production. However, the complexity of the gesture produced by the trainer in combination with the complexity of the target phoneme affected L2 learners’ success. Only when producing the less complex phoneme /u/, did participants benefit from seeing a more complex, iconic, gesture, making the AV-I condition the one in which L2 learners were most likely to learn. Conversely, when aiming to produce the more challenging phoneme /θ/, seeing a more complex gesture was actually detrimental to L2 learners, resulting in less learning taking place than in all other conditions. Additionally, the analysis corroborates our theoretical predictions concerning the complexity level of both phonemes. L2 learners often tended to already produce /u/ in a target-like way during the pretest, whereas they generally continued to be unable to correctly produce /θ/ during the posttest. This confirms that /u/ inherently is a less complex phoneme for Dutch learners of Spanish than /θ/.

## Study II

### Method

#### Participants

For this study, the data of 103 Spanish natives was analyzed. They were from the center of Spain; either from the autonomous region of Madrid, Castilla-La Mancha, or Castilla-León. On average, they were 30.9 years old (*SD* = 6.6 years) and 52 of them were women. None of the participants had auditory impairments that could have affected participation in the experiment. Participants were recruited via Qualtrics (2020) and received a small monetary reward for their participation.

#### Design

The experiment had a within-subjects design in which participants listened to target words from the pretest and the posttest produced by a subset of the participants of Study I. The productions were taken from three out of the four experimental conditions of Study I: AV, AV-P, and AV-I. The AO condition was left out to reduce the length of the perception tasks for the participants and because it represented a less natural learning context; most L2 learners are taught in a classroom setting where they can see the teacher. All participants judged these words for both perception measures.

#### Materials

Participants listened to randomly ordered target words produced by participants of study I. Because it was not feasible to have participants in study II to listen to all target words from experiment I, a selection was made. We used 8 items (2 with /u/ and 2 with /θ/, from both the pretest and the posttest) from 21 randomly selected speakers of study I. To make the experiment as interesting as possible for participants, the selected items were not the same ones for every speaker (e.g., the productions of *muro, nube, zeta*, and *zorro* as produced during the pretest and posttest were taken from one speaker, and productions of *ruta, suma, zueco*, and *zumo* as produced during the pretest and posttest were taken from another speaker). In total, participants listened to 168 items per measure (7 speakers × 2 items per phoneme × 2 phonemes × 3 gesture conditions × 2 testing moments).

#### Instruments

In separate blocks, participants judged each of the 168 items on accentedness and comprehensibility. Based on Derwing and Munro (1997), accentedness was measured with the statement “This person speaks …”, followed by a 7-point semantic differential anchored by “with a strong foreign accent – without a strong foreign accent” and comprehensibility was measured with the statement “This person is…”, followed by a 7-point semantic differential anchored by “Very hard to understand – very easy to understand”.

#### Procedure

The entire experiment took place online, in Spanish. Participants were given information about the experiment, and a consent form to sign, after which they filled out a short questionnaire on their language background. Subsequently, they performed the Spanish LexTALE task (Izura et al., 2014), which is a measure of Spanish vocabulary size. This enabled us to check that they were taking the task seriously, because, as native speakers, they should all be able to generate a high LexTALE score. Hence, any participants that performed below the L1 threshold of 47 points in the test were excluded from the final analysis. In the main part of the experiment, participants were randomly assigned to a block to rate one measure (either accentedness or comprehensibility), followed by a block rating the other measure. The entire experiment took about 30 min to complete.

### Results

Using R and the *psych* package (Revelle, 2019), the intra-class correlation coefficient was computed to assess the agreement between participants in rating the accentedness and comprehensibility of the words produced by our Dutch learners of Spanish in Study I. For both accentedness and comprehensibility, there was an excellent absolute agreement between the participants, using the two-way random effect models and “single rater” unit, both κ = 0.94, *p* < 0.05, which implies that they strongly agreed amongst themselves in regards to the accentedness and comprehensibility of the speech fragments that they listened to. In what follows, we will first report the data descriptively, followed by a report of the inferential statistics in the form of ordinal regression analyses for both measures.

**FIGURE 5:**
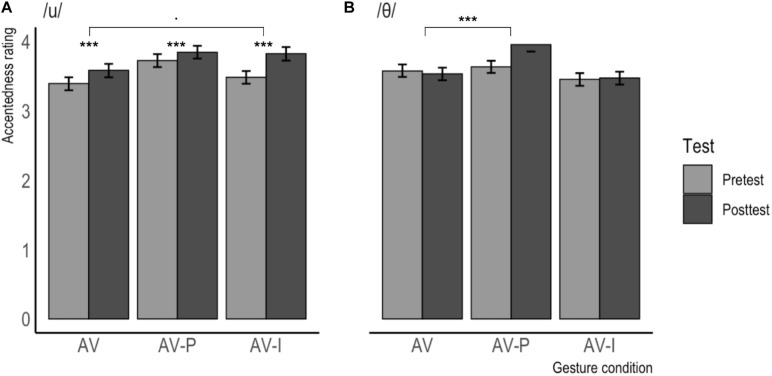
Mean accentedness ratings for /u/ **(A)** and /θ/ **(B)** produced at Pretest and Posttest, separated by gesture condition. Error bars represent confidence intervals. Higher scores indicate a less strong foreign accent.

The accentedness ratings per gesture condition (separated by phoneme) are visualized in Figure 5 and Table 3 contains the descriptive statistics per testing moment and gesture condition (split by phoneme), for accentedness. As can be seen from these results, the effects found in production appear to hold for perception as well: For items containing /u/, the difference between pre- and posttest is largest in the AV-I condition, whereas, for items containing /θ/, this difference is virtually non-existent in the AV-I condition, but largest in the AV-P condition.

**TABLE 3:** Accentedness ratings per predictor for items containing /u/ and /θ/.

Testing Moment	Gesture Condition	/u/		/θ/	
		*Mean*	*Standard deviation*	*Mean*	*Standard deviation*
Pretest	AV	3.39	1.81	3.58	1.74
	AV-P	3.72	1.81	3.63	1.69
	AV-I	3.48	1.76	3.45	1.77
Posttest	AV	3.58	1.88	3.53	1.75
	AV-P	3.84	1.78	3.95	1.83
	AV-I	3.82	1.89	3.47	1.79

**FIGURE 6:**
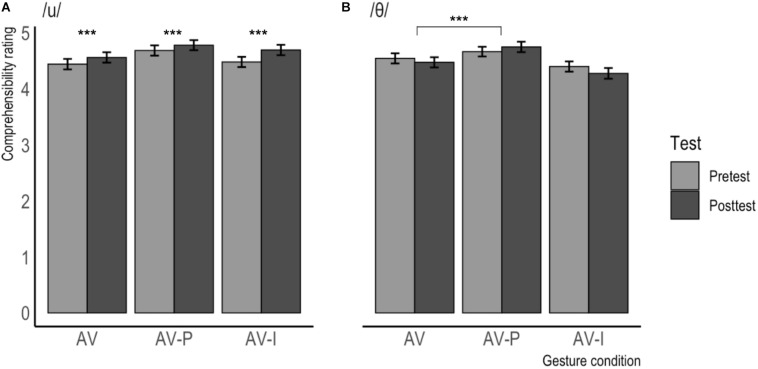
Mean comprehensibility ratings for /u/ **(A)** and /θ/ **(B)** produced at pretest and posttest, separated by gesture condition. Error bars represent confidence intervals. Higher scores indicate higher comprehensibility.

The comprehensibility ratings per gesture condition (separated by phoneme) are visualized in Figure 6 and Table 4 contains the descriptive statistics per testing moment and gesture condition, split by phoneme. In general, it can be noted that the comprehensibility ratings are roughly one scale point higher than the accentedness ratings. For items containing /u/, the difference between pre- and posttest is again largest in the AV-I condition, whereas for items containing /θ/, speakers who were in the AV-I condition were judged more difficult to comprehend after training than before, while they were deemed slightly easier to understand after training if they had been in the AV-P condition.

**TABLE 4:** Comprehensibility ratings per predictor for items containing /u/ and /θ/.

Testing Moment	Gesture Condition	/u/		/θ/	
		*Mean*	*Standard deviation*	*Mean*	*Standard deviation*
Pretest	AV	4.44	1.81	4.54	1.76
	AV-P	4.68	1.77	4.66	1.68
	AV-I	4.48	1.77	4.40	1.76
Posttest	AV	4.56	1.81	4.47	1.77
	AV-P	4.78	1.74	4.75	1.80
	AV-I	4.69	1.80	4.28	1.86

We will now evaluate the statistical evidence for the findings described above, which were based on visual inspection. We fitted ordinal regression models with random effects on the data for accentedness and comprehensibility separately, using R and the clmm function from the *ordinal* package (version 12-10, Christensen, 2019). We included the theoretically relevant predictors in the model: Testing Moment (pretest or posttest), Gesture Condition (AV, AV-P, or AV-I), Phoneme (/u/ or /θ/), and random intercepts by Participant, Speaker, and Item. Adding random slopes resulted in models that either failed to converge or had inferior fit. Significance was assessed via likelihood ratio tests comparing the full model to a model lacking only the relevant effect. The complete model provided the best fit as determined by the Akaike Information Criterion, see Tables 5, 6.

**TABLE 5:** Estimated effects and coefficients for accentedness ratings.

Predictor		*β* estimate	Std. error	*z* value	*p* value
Testing Moment_POSTTEST_		−0.055	0.066	−0.830	0.406
Gesture Condition_AV–P_		0.066	0.223	0.295	0.768
Gesture Condition_AV–I_		−0.058	0.225	−0.256	0.798
Phoneme_/u/_		−0.139	0.356	−0.390	0.696
**Testing Moment_POSTTEST_** * **Gesture Condition_AV–P_**		**0.428**	**0.093**	**4.478**	**0.000**
Testing Moment_POSTTEST_ * Gesture Condition_AV–I_		0.086	0.093	0.917	0.359
**Testing Moment_POSTTEST_** * **Phoneme_/u/_**		**0.284**	**0.094**	**3.018**	**0.003**
**Gesture Condition_AV–P_** * **Phoneme_/u/_**		**0.337**	**0.093**	**3.608**	**0.000**
Gesture Condition_AV–I_ * **Phoneme**_/u/_		0.050	0.094	0.527	0.598
**Testing Moment_POSTTEST_** * **Gesture Condition_AV–P_** * **Phoneme_/u/_**		−**0.492**	**0.133**	−**3.704**	**0.000**
Testing Moment_POSTTEST_ * Gesture Condition_AV–I_ * **Phoneme**_/u/_		0.074	0.133	0.559	0.576

**Random effects**	**Variance**	**Standard deviation**			

Participant	1.225	1.107			
Speaker	0.159	0.399			
Item	0.245	0.495			

*The intercept represents the following combination of variable levels: Testing Moment = Pretest, Gesture Condition = AV, Phoneme = /θ/. Asterisks (^∗^) represent interactions, subscript signals the level of a categorical variable. Significant *p*-values are printed in bold. The model used in this analysis can be described as: *Rating ∼ Testing Moment* × *Gesture Condition* × *Phoneme + (1| Participant) + (1 | Speaker) + (1 | Item)*.*

**TABLE 6:** Estimated effects and coefficients for comprehensibility ratings.

Predictor			*β* estimate	Std. error	*z* value	*p* value
Testing Moment_POSTTEST_			−0.098	0.066	−1.480	0.139
Gesture Condition_AV–P_			0.093	0.215	0.434	0.664
Gesture Condition_AV–I_			−0.070	0.216	−0.324	0.746
Phoneme_/u/_			−0.012	0.363	−0.034	0.973
**Testing Moment_POSTTEST_** * **Gesture Condition_AV–P_**			**0.264**	**0.094**	**2.802**	**0.005**
Testing Moment_POSTTEST_ * Gesture Condition_AV–I_			−0.031	0.094	−0.331	0.740
**Testing Moment_POSTTEST_** * **Phoneme_/u/_**			**0.262**	**0.094**	**2.783**	**0.005**
**Gesture Condition_AV–P_** * **Phoneme_/u/_**			**0.216**	**0.094**	**2.305**	**0.021**
Gesture Condition_AV–I_ * **Phoneme**_/u/_			−0.004	0.094	−0.046	0.963
**Testing Moment_POSTTEST_** * **Gesture Condition_AV–P_** * **Phoneme_/u/_**			−**0.313**	**0.134**	−**2.335**	**0.020**
Testing Moment_POSTTEST_ * Gesture Condition_AV–I_ * Phoneme_/u/_			0.128	0.133	0.962	0.336

**Random effects**	**Variance**	**Standard deviation**

Participant	1.756	1.325
Speaker	0.147	0.383
Item	0.255	0.505

*The intercept represents the following combination of variable levels: Testing Moment = Pretest, Gesture Condition = AV, Phoneme = /θ/. Asterisks (^∗^) represent interactions, subscript signals the level of a categorical variable. Significant *p*-values are printed in bold. The model used in this analysis can be described as: *Rating ∼ Testing Moment* × *Gesture Condition* × *Phoneme + (1| Participant) + (1| Speaker) + (1| Item)*.*

#### Accentedness

The ordinal regression analysis for accentedness revealed no main effects of Testing Moment, Gesture Condition or Phoneme, but several significant interactions were found, see Table 5. The analysis revealed a significant interaction effect between Testing Moment and Gesture Condition, with a bigger difference between the ratings at Pretest and Posttest in the AV-P condition (Pretest: *M* = 3.68, *SD* = 1.75; Posttest: *M* = 3.89, *SD* = 1.81; *M*Δ = 0.21) than in the AV condition (Pretest: *M* = 3.48, *SD* = 1.78; Posttest: *M* = 3.55, *SD* = 1.81; *M*Δ = 0.07). In addition, a significant interaction was found between Testing Moment and Phoneme, with a bigger difference between the ratings at Pretest and Posttest for the items containing /u/ (Pretest: *M* = 3.53, *SD* = 1.80; Posttest: *M* = 3.75, *SD* = 1.85; *M*Δ = 0.22) than for those containing /θ/ (Pretest: *M* = 3.55, *SD* = 1.74; Posttest: *M* = 3.65, *SD* = 1.80; *M*Δ = 0.10). The analysis also revealed a significant interaction effect between Gesture Condition and Phoneme, with a bigger difference between the AV and AV-P conditions for the items containing /u/ (AV: *M* = 3.48, *SD* = 1.84; AV-P: *M* = 3.78, *SD* = 1.80; *M*Δ = 0.30) than for those containing /θ/ (AV: *M* = 3.55, *SD* = 1.75; AV-P: *M* = 3.79, *SD* = 1.77; *M*Δ = 0.24).

Finally, the model revealed a three-way interaction between Testing Moment, Phoneme, and Gesture condition. In order to interpret this interaction, we performed two separate mixed ordinal regression analyses, one on items containing /u/ and one on items containing /θ/. These analyses show that the above-mentioned interaction between Testing Moment_POSTTEST_ and Gesture Condition_AV–P_ was significant for the items containing /θ/ (β = 0.434, *SE =* 0.094, *z* = 4.628, *p* < 0.001), but not significant for the items containing /u/ (β = −0.062, *SE* = 0.094, *z* = −0.660, *p* = 0.509). For items containing /θ/, there was a bigger difference between the ratings at Pretest and Posttest in the AV-P condition (Pretest: *M* = 3.63, *SD* = 1.69; Posttest: *M* = 3.95, *SD* = 1.83; *M*Δ = 0.32) than in the AV condition (Pretest: *M* = 3.58, *SD* = 1.74; Posttest: *M* = 3.53, *SD* = 1.75; *M*Δ = −0.05). For items containing /u/, there was no difference between the ratings at Pretest and Posttest in the AV-P condition (Pretest: *M* = 3.72, *SD* = 1.81; Posttest: *M* = 3.84, *SD* = 1.78; *M*Δ = 0.12) and the AV condition (Pretest: *M* = 3.39, *SD* = 1.81; Posttest: *M* = 3.58, *SD* = 1.88; *M*Δ = 0.19). In addition, the analysis on the items containing /u/ revealed a trend for the interaction between Testing Moment_POSTTEST_ and Gesture Condition_AV–I_ (β = 0.176, *SE* = 0.095, *z* = 1.856, *p* = 0.063) in which there was a bigger difference between the ratings at Pretest and Posttest in the AV-I condition (Pretest: *M* = 3.48, *SD* = 1.76; Posttest: *M* = 3.82, *SD* = 1.89; *M*Δ = 0.34) than in the AV condition (Pretest: *M* = 3.39, *SD* = 1.81; Posttest: *M* = 3.58, *SD* = 1.88; *M*Δ = 0.19). Finally, the analysis on the items containing /u/ also revealed a significant main effect of Testing Moment, with higher ratings at Posttest (*M* = 3.75, *SD* = 1.85) than at Pretest (*M* = 3.53, *SD* = 1.80), irrespective of Gesture Condition.

#### Comprehensibility

For comprehensibility, the ordinal regression analysis also revealed no significant main effects, but several significant interactions between the fixed factors were found. The analysis revealed a significant interaction effect between Testing Moment and Gesture Condition, with a bigger difference between the ratings at Pretest and Posttest in the AV-P condition (Pretest: *M* = 4.67, *SD* = 1.72; Posttest: *M* = 4.76, *SD* = 1.77; *M*Δ = 0.09) than in the AV condition (Pretest: *M* = 4.49, *SD* = 1.79; Posttest: *M* = 4.52, *SD* = 1.79; *M*Δ = 0.03). In addition, a significant interaction was found between Testing Moment and Phoneme, with a bigger difference between the ratings at Pretest and Posttest for the items containing /u/ (Pretest: *M* = 4.53, *SD* = 1.79; Posttest: *M* = 4.68, *SD* = 1.78; *M*Δ = 0.15) than for those containing /θ/ (Pretest: *M* = 4.53, *SD* = 1.74; Posttest: *M* = 4.50, *SD* = 1.82; *M*Δ = −0.03). The analysis also revealed a significant interaction effect between Gesture Condition and Phoneme, with a bigger difference between the AV and AV-P conditions for the items containing /u/ (AV: *M* = 4.50, *SD* = 1.81; AV-P: *M* = 4.73, *SD* = 1.76; *M*Δ = 0.23) than for those containing /θ/ (AV: *M* = 4.51, *SD* = 1.76; AV-P: *M* = 4.71, *SD* = 1.74; *M*Δ = 0.20).

Finally, the model revealed a three-way interaction between Testing Moment, Phoneme, and Gesture condition. In order to interpret this interaction, we performed two separate mixed ordinal regression analyses, one on items containing /u/ and one on items containing /θ/. These analyses show that the above-mentioned interaction between Testing Moment_POSTTEST_ and Gesture Condition_AV–P_ was significant for the items containing /θ/ (β = 0.268, *SE =* 0.094, *z* = 2.843, *p* < 0.01) but not significant for the items containing /u/ (β = −0.037, *SE* = 0.095, *z* = −0.384, *p* = 0.701). For items containing /θ/, there was a bigger difference between the ratings at Pretest and Posttest in the AV-P condition (Pretest: *M* = 4.66, *SD* = 1.68; Posttest: *M* = 4.75, *SD* = 1.80; *M*Δ = 0.09) than in the AV condition (Pretest: *M* = 4.54, *SD* = 1.76; Posttest: *M* = 4.47, *SD* = 1.77; *M*Δ = −0.07). For items containing /u/, there was no difference between the ratings at Pretest and Posttest in the AV-P condition (Pretest: *M* = 4.68, *SD* = 1.77; Posttest: *M* = 4.78, *SD* = 1.74; *M*Δ = 0.10) and in the AV condition (Pretest: *M* = 4.44, *SD* = 1.81; Posttest: *M* = 4.56, *SD* = 1.81; *M*Δ = 0.12). In addition, the analysis on the items containing /u/ revealed a significant main effect of Testing Moment, with higher ratings at Posttest (*M* = 4.68, *SD* = 1.78) than at Pretest (*M* = 4.53, *SD* = 1.79), irrespective of Gesture Condition.

### Interim Discussion

In summary, Study II showed that the findings of Study I, in which the more complex iconic gesture facilitated the production of the less complex phoneme /u/ but not the production of the more complex phoneme /θ/, and the less complex pointing gesture facilitated the production of the more complex phoneme /θ/ but less so for the production of the less complex phoneme /u/, were confirmed. When a pointing gesture was included in the training, this was particularly helpful for items containing /θ/, but not for items containing /u/, resulting in less foreign-accentedness and higher perceived comprehensibility for /θ/ items. For items containing /u/, seeing an iconic gesture during training lead to speech being judged as less foreign-accented but equally comprehensible. Also, again in line with the findings from Study I, Study II showed that /u/ was easier and /θ/ was harder to acquire; scores on foreign-accentedness and perceived comprehensibility differed more between the pretest and posttest for /u/ than for /θ/. Although these results show that the interaction between type of gesture and type of phoneme during training affects perceived accentedness and comprehensibility, we should realize that the effects were relatively small; the differences in scores between pretest and posttest were generally less than one point on a 7-point scale. Finally, we found that the ratings for accentedness were lower than the ratings for comprehensibility. As found in previous work, it appears that although native listeners are sensitive to hearing deviations from native pronunciation, this does not necessarily result in a lower comprehensibility score (Derwing and Munro, 1997; Munro and Derwing, 1999; Van Maastricht et al., 2016, 2020).

## General Discussion

The goal of this study was to investigate if gestures can facilitate L2 phoneme acquisition, and, more specifically, in what way the complexity of the gesture and the complexity of the phoneme play a role in this process. We focused on the acquisition of two Spanish phonemes which are typically hard for native speakers of Dutch: /u/ and /θ/. We expected /u/ to be easier to acquire because, although the grapheme it is typically associated with in Dutch differs from the grapheme typically used in Spanish, the phoneme /u/ does also occur in the Dutch phoneme inventory. We expected /θ/ to be harder to acquire because, in addition to the Spanish grapheme associated with this phoneme being pronounced differently in Dutch, the phoneme is not part of the Dutch phoneme inventory. We hypothesized that adding audio-visual information to the phoneme training that Dutch learners of Spanish received would facilitate phoneme acquisition, as compared to providing only audio information. In addition, we expected that including gestures in the phoneme training would be most beneficial for phonemes that are less cognitively demanding, in this case /u/, rather than /θ/. Phoneme training took place in one of four conditions: audio-only, audio-visual, audio-visual with a pointing gesture, or audio-visual with an iconic gesture. Given the lack of previous studies comparing the effect of different types of gestures on phoneme acquisition, we did not have clear predictions concerning which type of gesture would work best. Based on the idea that processing a pointing gesture is less cognitively demanding than processing an iconic gesture, we speculated that a pointing gesture might be more helpful than an iconic gesture during phoneme acquisition because processing a cognitively less demanding pointing gesture would leave more processing resources available for the perception and acquisition of the new phoneme, as compared to processing a cognitively more demanding iconic gesture. We conducted two studies to investigate these issues: Study I, in which native speakers of Dutch received training in one of the four conditions and produced the Spanish target phonemes in a pretest and posttest, and Study II, in which native speakers of Spanish listened to the words containing the target phonemes as produced by the Dutch learners of Spanish before and after training and scored these on accentedness and comprehensibility.

The results of both studies showed that, in general, adding audio-visual information to phoneme pronunciation training facilitates target-like production. However, it matters which gesture is added to the training of which phoneme, as the specific gesture-phoneme combination can result in more, or less, target-like production, accentedness, and perceived comprehensibility. Also, the results of both studies complement each other in the sense that the improvements in phoneme production in certain experimental conditions in Study I were generally reflected in less foreign-accentedness and higher comprehensibility ratings for items from these same conditions in Study II.

Returning to our hypotheses, we find that our data confirm our first prediction, namely that /u/ would be easier to acquire than /θ/ for Dutch learners of Spanish. Study I showed that /u/ was often already produced in a target-like manner during the pretest, whereas /θ/ was often never produced in a target-like manner, regardless of training condition. Study II also showed that between the pretest and posttest items containing /u/ were rated as less foreign-accented and more comprehensible, which was not the case for items containing /θ/. These findings suggest that if /u/ had not already been acquired before training, it can be acquired during training, but that in many cases, a single training session is not sufficient to benefit the acquisition of /θ/.

With respect to our second hypothesis, we find partial corroboration of earlier work (Hardison, 2003; Hazan et al., 2005) in our results. Study I revealed that adding audio-visual information to training that includes an iconic gesture affects target-like production, as compared to providing audio-only information. Whether this effect on target-like production is positive or negative depends on the phoneme in question: If the phoneme being acquired was /u/, seeing an iconic gesture during training led to more cases of learning. However, if the phoneme being acquired was /θ/, seeing an iconic gesture during training was detrimental, leading to fewer cases of learning than in all other conditions. In other words, seeing a complex gesture during training facilitated the target-like production of the easy phoneme, whereas seeing a complex gesture during training harmed the target-like production of the complex phoneme. The importance of the phoneme-gesture combination is also reflected in the results of Study II: The specific gesture being used during training could result in less foreign-accentedness and higher comprehensibility, but this depended on which phoneme was being produced. Seeing a less complex pointing gesture during training lead to productions of words with /θ/ that were perceived as less foreign-accented and more comprehensible. Seeing a more complex iconic gesture during training lead to productions of words with /u/ that were perceived as less foreign-accented. This means that our speculation, based on findings by Kelly et al. (2017), that the less cognitively demanding pointing gesture would facilitate acquisition most, was not supported by all the data: The facilitative effect of the pointing gesture depended on which phoneme was being acquired: pointing gestures worked best for the complex phoneme /θ/, but not for the easy phoneme /u/. The more complex iconic gesture helped in the acquisition of the easier phoneme /u/, but hindered acquisition of /θ/.

These results mean that the complexity of both the gesture and the phoneme matters when using gesture in L2 phoneme acquisition. It appears that a complex phoneme is best combined with a simple gesture, and the other way around. Most previous studies did not take the complexity of the target phoneme or gesture into account, and this may help to explain some of the contradictory prior findings. For instance, Kelly et al. (2014) investigated the effect of metaphoric gestures in the context of Japanese vowel length contrasts as perceived by American learners. While their study mainly revealed no differences between the learners that had seen or seen and produced gestures during training, they reported one case in which there was a significant difference between their experimental groups. While half of their participants were trained on metaphoric gestures representing the vowel length as a syllable, the other half was trained on gestures representing the vowel length as a mora. Reaction times of participants from the latter group were significantly longer than those of participants in the former group during an auditory identification test, which implies that American learners needed more time to process the gesture related to the mora category, which is non-native to them, than the gesture related to the syllable category, which is native to them. This is in line with the findings of the current study, because a gesture representing an unfamiliar phonemic element (mora) is arguably more complex for L2 learners than a gesture representing a familiar phonemic element (syllable).

Moreover, the current results are in line with the idea proposed by Kelly and Lee (2012) that gesture may only help when the task demands are not too high. In their work, they focused on L2 vocabulary learning and distinguished between phonetically easy and phonetically hard word pairs. Their results showed that (iconic) gestures helped for the easy word pairs, but actually hindered the vocabulary acquisition for the hard word pairs. Importantly, in their work, they did not distinguish between types of gestures but only used iconic gestures, and with the results they found they wonder whether it may be the case that gestures that convey less or no semantic meaning, as compared to iconic gestures, would also hinder acquisition, or whether the fact that iconic gestures carry semantic information is a reason for the fact that they do not always help, and may even hinder the learner. The pointing gesture used in our current study is an example of such a gesture that conveys less semantic information. After all, a pointing gesture mainly serves as a manual highlighter and, at least in the current study, does not provide any information about the speech it accompanies. If we contrast this with the iconic gestures used in our study we can see that those contained quite a bit of semantic information, more specifically, the gestures visualized what the relevant articulators were of the specific phoneme, and how these articulators should be used to produce the phoneme. This suggests that seeing the iconic gesture cost a fair amount of processing energy, as compared to seeing the pointing gesture. This processing energy may come at a cost to the resources that are left for focusing on listening to the sound of the phoneme and watching the actual articulators needed for phoneme production. If more cognitive energy is needed because the phoneme in question does not exist in the native language phoneme inventory, this may result in less, rather than more, acquisition taking place. Likewise, if the processing of the phoneme takes less cognitive effort, for example, because the phoneme is already familiar, there is more processing space left to take the gesture that is being produced into account. Again, this is in line with the suggestion given by Kelly and Lee (2012) that gesture may only facilitate the processing of sounds that are familiar in someone’s native language.

Naturally, our findings can be expanded on in several ways. More studies are needed to further determine which elements of a gesture or phoneme contribute to its complexity. Specifically, it would be interesting to compare different types of gestures on only one dimension. The complex gestures used for the two phonemes in our study were both iconic in nature, but the one visualizing the articulator needed to produce /u/ highlighted the lips, whereas the one representing the articulator involved in the production of /θ/ highlighted the tongue. Comparing two phonemes that are more similar in their articulation but different with respect to their presence in the L1 inventory, such as /a/ and /ɑ/ for a native speaker of Spanish, would enable a comparison of two iconic gestures that represent the same articulators and that thus are more similar in form. Of course, this also generates a challenge: if the articulation of two phonemes is very similar, can the two gestures reflect the relevant information and remain sufficiently different to be useful? In the same vein, more different types of (less complex) gestures should be compared, for instance, beats versus pointing gestures. It might well be the case that gesture complexity is not so much a dichotomous concept, but rather one that spans a continuum.

Similarly, we have defined phoneme complexity in our study as the extent to which our participants were familiar with the used phonemes in their L1, but it seems reasonable to assume that other factors contribute to a phoneme’s complexity. A comparison of two phonemes that are both not included in the participants’ L1 inventory, but that differ in the necessary articulators, might generate more insight in whether phoneme complexity, like gesture complexity, is a continuum on which “presence in the L1 inventory” might be of more importance than “familiarity with the articulators.” For instance, one could compare the uvular /χ/ and glottal /ɦ/, both of which are typical of Dutch but do not occur in the French phoneme inventory. While the French phoneme inventory does contain another uvular phoneme, /ʁ/, there are no other glottals in the system, which might make /ɦ/ a more difficult sound to acquire for French natives than /χ/. In addition, the effect of gesture and sound complexity might not only hold for segmental sounds but could also apply to suprasegmentals, as implied by the results of Kelly et al. (2017). Finally, the relative weight of certain segments in communication between L1 and L2 speakers may also be of consideration in this respect. As shown for English by Suzukida and Saito (2019), the Functional Load principle (as applied to L2 pronunciation teaching by Brown, 1988) can be used as a tool to determine which segments are crucial for successful understanding in L1-L2 communication.

A potential limitation to the current study is that participants in Study I received only one short training, and were tested almost immediately after this training. This means that we do not know to what extent the current results also apply to long term learning and whether repeated training yields different results. Results obtained by Zhen et al. (2019) and Li et al. (2020), which had similarly, short training sessions (of seven and two and a half minutes, respectively), imply that it is possible to obtain effects from only one short training, even long term. The fact that we found effects of gesture and phoneme complexity on the acquisition of L2 phonemes after only one short training corroborate their findings and are promising in the sense that we expect more or longer training to strengthen our results. Another potential limitation is that Study I took place in a laboratory setting, in which participants took part individually in a soundproof booth. Although this meant that we were able to control the experimental conditions and receive high-quality sound recordings, it also means that the external validity of this study is restricted, in the sense that the laboratory setting was not representative of a classroom setting in which pronunciation training may normally take place.

In conclusion, more research is needed in the context of the possibly beneficial role of gestures in foreign language acquisition and the role of complexity in this context. Prior, present, and future results in this context do not only further inform the theory regarding the nature of multimodal communication and (foreign) language learning, but are also directly relevant in practice. In (foreign) language acquisition, but also in many other fields, knowing which gesture works in which context is crucial. For example, an educational method that is currently popular in primary schools in the Netherlands encourages teachers and pupils to use gestures to facilitate the coupling of segments and graphemes in reading development. While it might well be the case that gestures can be helpful in this context, the types of gestures used range from iconic to metaphoric and even enactment gestures, which might influence their efficacy. In learning more about how gesture and phoneme complexity influence the efficacy of gestures in the context of L2 phoneme acquisition, we have made a start in discovering just how handy gestures can be.

## Data Availability Statement

The raw data supporting the conclusion of this article will be made available by the authors, without undue reservation.

## Ethics Statement

The studies involving human participants were reviewed and approved by Ethics Assessment Committee Humanities, Radboud University. The patients/participants provided their written informed consent to participate in this study. Written informed consent was obtained from the individual(s) for the publication of any potentially identifiable images or data included in this article.

## Author Contributions

Both authors contributed equally to the conception and design of the study, the revision of the manuscript, and read and approved the submitted version. LM performed the statistical analyses and wrote the first draft of the sections “Method” and “Results.” MH wrote the first draft of the sections “Introduction” and “Discussion.”

## Conflict of Interest

The authors declare that the research was conducted in the absence of any commercial or financial relationships that could be construed as a potential conflict of interest.

## Footnotes

1 Alternatively, the lack of effect in the perception task may also be due to a ceiling effect (cf. Hayes-Harb and Masuda, 2008; Li et al., 2020).

2 While /θ/ is not included in the Dutch phoneme inventory, it is not the case that our participants are completely unfamiliar with this phoneme given that the participants of the current study, who are for the most part university students, will have all had at least 6 years of English education. However, /θ/ is a notoriously difficult phoneme for L1 speakers of Dutch in English as well (Gussenhoven and Broeders, 1997; Collins and Mees, 2003; Van den Doel, 2006; Hanulíková and Weber, 2012) and there are subtle differences in the phonetic realization of /θ/ in English (dental) and Spanish (interdental) that are still to be acquired.

3 We are aware that the grapheme <c> is also pronounced as /θ/ in certain contexts in Castilian Spanish, but have chosen not to include this grapheme in our design in order to not overcomplicate the task for the novice Dutch learners of Spanish who participated in our production task.

4 In theory, 400 comparisons can be made between the performance at pretest and posttest (4 items × 2 segments × 50 participants × 2 moments = 800 productions, which equals 400 comparisons). In practice, 8 data points were lost due to inferior sound quality or coding difficulties. Per training condition, the maximally possible total is 96 for AO and AV-I and 104 for AV and AV-P. For relative frequencies in the form of percentages, see Figure 4.

## References

[B1] Bates D. Mächler M. Bolker B. Walker S. (2015). Fitting linear mixed-effects models using lme4. *J. Stat. Softw.* 67 1–48. 10.18637/jss.v067.i01

[B2] Boersma P. Weenink D. (2018). *Praat: Doing Phonetics by Computer (Version 6.0.49) [Computer Program].* Available online at: http://www.praat.org/ (accessed March 1, 2020).

[B3] Bohn O.-S. Munro M. J. (eds) (2007). *Language Experience in Second Language Speech Learning: In Honor of James Emil Flege.* Amsterdam: John Benjamins.

[B4] Brown A. (1988). Functional load and the teaching of pronunciation. *TESOL Q.* 22 593–606. 10.2307/3587258

[B5] Caspers J. Horłoza K. (2012). Intelligibility of non-natively produced Dutch words: interaction between segmental and suprasegmental errors. *Phonetica* 69 94–107. 10.1159/000342622 23172241

[B6] Christensen R. H. B. (2019). *Ordinal—Regression Models for Ordinal Data (Version R Package Version 2019.12-10.).* Available online at: https://CRAN.R-project.org/package=ordinal (accessed December 10, 2019).

[B7] Collins B. Mees I. (2003). *The Phonetics of English and Dutch, Revised Edition.* Leiden, NY: Brill.

[B8] Derwing T. (2003). What do ESL students say about their accents? *Can. Mod. Lang. Rev.* 59 547–567. 10.3138/cmlr.59.4.547

[B9] Derwing T. Munro M. (1997). Accent, intelligibility, and comprehensibility: evidence from four L1s. *Stud. Second Lang. Acquis.* 20 1–16. 10.1017/s0272263197001010

[B10] Derwing T. Munro M. (2009). Comprehensibility as a factor in listener interaction preferences: implications for the workplace. *Can. Mod. Lang. Rev.* 66 181–202. 10.3138/cmlr.66.2.181

[B11] Escudero P. Simon E. Mulak K. E. (2014). Learning words in a new language: orthography doesn’t always help. *Biling. Lang. Cogn.* 17 384–395. 10.1017/s1366728913000436

[B12] Gluhareva D. Prieto P. (2017). Training with rhythmic beat gestures benefits L2 pronunciation in discourse-demanding situations. *Lang. Teach. Res.* 21 609–631. 10.1177/1362168816651463

[B13] Goldin-Meadow S. (2005). *Hearing Gesture: How Our Hands Help us Think.* Cambridge, MA: The Belknap Press.

[B14] Goldin-Meadow S. Kim S. Singer M. (1999). What the teacher’s hands tell the student’s mind about math. *J. Educ. Psychol.* 91 720–730. 10.1037/0022-0663.91.4.720

[B15] Graziano M. Gullberg M. (2018). When speech stops, gesture stops: evidence from developmental and crosslinguistic comparisons. *Front. Psychol.* 9:879. 10.3389/fpsyg.2018.00879 29910761 PMC5992892

[B16] Gullberg M. (2006). Some reasons for studying gesture and second language acquisition (hommage a Adam Kendon). *Int. Rev. Appl. Linguist.* 44 103–124.

[B17] Gussenhoven C. H. M. Broeders A. P. A. (1997). *English Pronunciation for Student Teachers.* Winschoterdiep: Noordhoff Uitgevers.

[B18] Hannah B. Wang Y. Jongman A. Sereno J. A. (2017). Cross-modal association between auditory and visual-spatial information in Mandarin tone perception. *J. Acoust. Soc. Am.* 140 3225–3225. 10.1121/1.4970187 PMC5722845 29255435

[B19] Hanulíková A. Weber A. (2012). Sink positive: linguistic experience with th substitutions influences nonnative word recognition. *Atten. Percept. Psychophys.* 74 613–629. 10.3758/s13414-011-0259-7 22207311

[B20] Hardison D. (2003). Acquisition of second-language speech: effects of visual cues, context, and talker variability. *Appl. Psycholinguist.* 24 495–522. 10.1017/s0142716403000250

[B21] Hayes-Harb R. Masuda K. (2008). Development of the ability to lexically encode novel second language phonemic contrasts. *Second Lang. Res.* 24 5–33. 10.1177/0267658307082980

[B22] Hazan V. Sennema A. Iba M. Faulkner A. (2005). Effect of audiovisual perceptual training on the perception and production of consonants by Japanese learners of English. *Speech Commun.* 47 360–378. 10.1016/j.specom.2005.04.007

[B23] Hirata Y. Kelly S. Huang J. Manansala M. (2014). Effects of hand gestures on auditory learning of second-language vowel length contrasts. *J. Speech Lang. Hear. Res.* 57 2090–2101. 10.1044/2014_jslhr-s-14-0049 25088127

[B24] Hoetjes M. Van Maastricht L. Van der Heijden L. (2019a). “Gestural training benefits L2 phoneme acquisition: findings from a production and perception perspective,” in *Proceedings of the 6th Gesture and Speech in Interaction (GESPIN)*, ed. Grimminger A. (Paderborn: Universitatsbibliothek Paderborn), 50–55.

[B25] Hoetjes M. van Maastricht L. Van der Heijden L. (2019b). “Multimodal training can facilitate L2 phoneme acquisition,” in *Paper Presented at the EuroSLA*, Lund.

[B26] Izura C. Cuetos F. Brysbaert M. (2014). Lextale-Esp: a test to rapidly and efficiently assess the Spanish vocabulary size. *Psicologica* 35 49–66.

[B27] Kelly S. Bailey A. Hirata Y. (2017). Metaphoric gestures facilitate perception of intonation more than length in auditory judgments of non-native phonemic contrasts. *Collabra Psychol.* 3:7. 10.1525/collabra.76 33021500

[B28] Kelly S. Hirata Y. Manansala M. Huang J. (2014). Exploring the role of hand gestures in learning novel phoneme contrasts and vocabulary in a second language. *Front. Psychol.* 5:673. 10.3389/fpsyg.2014.00673 25071646 PMC4077026

[B29] Kelly S. Lee A. (2012). When actions speak too much louder than words: hand gestures disrupt word learning when phonetic demands are high. *Lang. Cogn. Process.* 27 793–807. 10.1080/01690965.2011.581125

[B30] Kelly S. McDevitt T. Esch M. (2009). Brief training with co-speech gesture lends a hand to word learning in a foreign language. *Lang. Cogn. Process.* 24 313–334. 10.1080/01690960802365567

[B31] Kendon A. (2004). *Gesture. Visible Action as Utterance.* Cambridge: Cambridge University Press.

[B32] Kooij J. Van Oostendorp M. (2003). *Fonologie: Uitnodiging tot de Klankleer van Het Nederlands.* Amsterdam: Amsterdam University Press.

[B33] Lev-Ari S. Keysar B. (2010). Why don’t we believe non-native speakers? The influence of accent on credibility. *J. Exp. Soc. Psychol.* 46 1093–1096. 10.1016/j.jesp.2010.05.025 27841010

[B34] Li P. Baills F. Prieto P. (2020). Observing and producing durational hand gestures facilitates the pronunciation of novel vowel-length contrasts. *Stud. Second Lang. Acquis.* (in press). 10.1017/S0272263120000054

[B35] Macedonia M. Mueller K. Friederici A. (2011). The impact of iconic gestures on foreign language word learning and its neural substrate. *Hum. Brain Mapp.* 32 982–998. 10.1002/hbm.21084 20645312 PMC6870319

[B36] Madan C. R. Singhal A. (2012). Using actions to enhance memory: effects of enactment, gestures, and exercise on human memory. *Front. Psychol.* 3:507. 10.3389/fpsyg.2012.00507 23293612 PMC3536268

[B37] McNeill D. (1992). *Hand and Mind. What Gestures Reveal About Thought.* Chicago, IL: University of Chicago Press.

[B38] Morett L. Chang L. Y. (2015). Emphasising sound and meaning: pitch gestures enhance Mandarin lexical tone acquisition. *Lang. Cogn. Neurosci.* 30 347–353. 10.1080/23273798.2014.923105

[B39] Munro M. J. Derwing T. M. (1999). Foreign accent, comprehensibility, and intelligibility in the speech of second language learners. *Lang. Learn.* 49 285–310. 10.1111/0023-8333.49.s1.8

[B40] Qualtrics (2020). *[Computer Software].* Available online at: http://www.qualtrics.com/ (accessed March 1, 2020).

[B41] RCoreTeam (2019). *R: A Language and Environment for Statistical Computing.* Vienna: R Foundation for Statistical Computing.

[B42] Revelle W. (2019). *psych: Procedures for Psychological, Psychometric, and Personality Research.* Evanston, IL: Northwestern University.

[B43] Seyfeddinipur M. (2006). *Disfluency: Interrupting Speech and Gesture.* Nijmegen: Radboud University Nijmegen.

[B44] Smotrova T. (2017). Making pronunciation visible: gesture in teaching pronunciation. *TESOL Q.* 51 59–89. 10.1002/tesq.276

[B45] Suzukida Y. Saito K. (2019). Which segmental features matter for successful L2 comprehensibility? Revisiting and generalizing the pedagogical value of the functional load principle. *Lang. Teach. Res.* (in press). 10.1177/1362168819858246

[B46] Tellier M. (2008). The effect of gestures on second language memorisation by young children. *Gesture* 8 219–235. 10.1075/gest.8.2.06tel

[B47] Timmis I. (2002). Native-speaker norms and International English: a classroom view. *ELT J.* 56 240–249. 10.1093/elt/56.3.240

[B48] Van den Doel R. (2006). *How Friendly are the Natives? An Evaluation of Native-Speaker Judgements of Foreign-Accented British and American English.* Amsterdam: Netherlands Graduate School of Linguistics.

[B49] Van Maastricht L. Hoetjes M. van der Heijden L. (2019). “Multimodal training facilitates L2 phoneme acquisition: an acoustic analysis of Dutch learners’ segment production in Spanish,” in *Proceedings of the 19th International Congress of Phonetic Sciences, Melbourne, Australia*, eds Calhoun S. Escudero P. Tabain M. Warren P. (Canberra: Australasian Speech Science and Technology Association Inc), 3528–3532.

[B50] Van Maastricht L. Krahmer E. Swerts M. (2016). Native speaker perceptions of (non-)native prominence patterns: effects of deviance in pitch accent distribution on accentedness, comprehensibility, intelligibility, and nativeness. *Speech Commun.* 83 21–33. 10.1016/j.specom.2016.07.008

[B51] Van Maastricht L. Zee T. Krahmer E. Swerts M. (2020). The interplay of prosodic cues in the L2: how intonation, rhythm, and speech rate in speech by Spanish learners of Dutch contribute to L1 Dutch perceptions of accentedness and comprehensibility. *Speech Commun.* (in press). 10.1016/j.specom.2020.04.003

[B52] Van Nispen K. van de Sandt-Koenderman W. M. E. Mol L. Krahmer E. (2016). Pantomime production by people with aphasia: what are influencing factors? *J. Speech Lang. Hear. Res.* 59 745–758. 10.1044/2015_JSLHR-L-15-0166 27387394

[B53] Wagner P. Malisz Z. Kopp S. (2014). Gesture and speech in interaction: an overview. *Speech Commun.* 57 209–232. 10.1016/j.specom.2013.09.008

[B54] Yeo A. Wagner Cook S. Nathan M. J. Popescu V. Alibali M. (2018). “Instructor gesture improves encoding of mathematical representation,” in *Proceedings of the 40th Annual Conference of the Cognitive Science Society*, eds Rogers T. T. Rau M. Zhu X. Kalish C. W. (Austin, TX: Cognitive Science Society), 2723–2728.

[B55] Zhang Y. Baills F. Prieto P. (2020). Hand-clapping to the rhythm of newly learned words improves L2 pronunciation: evidence from training Chinese adolescents with French words. *Lang. Teach. Res.* 24 666–689. 10.1177/1362168818806531

[B56] Zhen A. Van Hedger S. Heald S. Goldin-Meadow S. Tian X. (2019). Manual directional gestures facilitate cross-modal perceptual learning. *Cognition* 187 178–187. 10.1016/j.cognition.2019.03.004 30877849

